# Cost‐Effectiveness Analysis of Empagliflozin for Treatment of Patients With Heart Failure With Reduced Ejection Fraction in the United States

**DOI:** 10.1161/JAHA.123.029042

**Published:** 2024-02-16

**Authors:** Odette S. Reifsnider, Ali Tafazzoli, Stephan Linden, Jack Ishak, Pal Rakonczai, Matthew Stargardter, Effie Kuti

**Affiliations:** ^1^ Evidera Bethesda MD; ^2^ Boehringer Ingelheim International GmbH Ingelheim am Rhein Germany; ^3^ Evidera Budapest Hungary; ^4^ Boehringer Ingelheim Pharmaceuticals, Inc Ridgefield CT

**Keywords:** cost‐effectiveness, empagliflozin, heart failure, sodium–glucose co‐transporter 2 inhibitor, United States, Heart Failure, Pharmacology, Quality and Outcomes

## Abstract

**Background:**

In the EMPEROR‐Reduced trial (Empagliflozin Outcome Trial in Patients with Chronic Heart Failure and a Reduced Ejection Fraction), empagliflozin plus standard of care reduced the composite of cardiovascular death or hospitalization for heart failure versus standard of care in adults with heart failure with reduced ejection fraction. This analysis investigated the cost‐effectiveness of the 2 regimens from the perspective of US payors.

**Methods and Results:**

A Markov cohort model was developed based on Kansas City Cardiomyopathy Questionnaire Clinical Summary Score quartiles and death. Transition probabilities between health states, risk of cardiovascular/all‐cause death, hospitalization for heart failure and adverse events, treatment discontinuation, and health utilities were estimated from trial data. Medicare and commercial payment rates were combined for treatment acquisition, acute event management, and disease management. An annual discount rate of 3% was used. Empagliflozin plus standard of care yielded 18% fewer hospitalizations for heart failure and 6% fewer deaths versus standard of care over a lifetime, providing cost‐offsets while adding 0.19 life years and 0.19 quality‐adjusted life years at an incremental cost of $16 815/patient. The incremental cost‐effectiveness ratio was $87 725/quality‐adjusted life years gained. Results were consistent across payors, subpopulations, and in deterministic sensitivity analyses. In probabilistic sensitivity analyses, empagliflozin plus standard of care was cost‐effective in 3%, 62%, and 80% of iterations at thresholds of $50 000, $100 000, and $150 000/quality‐adjusted life years.

**Conclusions:**

Empagliflozin plus standard of care may prevent hospitalizations for heart failure, extend life, and increase quality‐adjusted life years for patients with heart failure with reduced ejection fraction at an acceptable cost for US payors.

**Nonstandard Abbreviations and Acronyms**
ARNi: angiotensin receptor neprilysin inhibitor
HFrEF: heart failure with reduced ejection fraction
HHF: hospitalization for heart failure
ICER: incremental cost‐effectiveness ratio
KCCQ‐CSS: Kansas City Cardiomyopathy Questionnaire Clinical Summary Score
SoC: standard of care

**Clinical Perspective**

**What Is New?**

- Cost‐effectiveness analysis based on EMPEROR‐Reduced trial (Empagliflozin Outcome Trial in Patients with Chronic Heart Failure and a Reduced Ejection Fraction) findings has shown that the addition of empagliflozin to standard of care relative to standard of care alone may prevent hospitalizations for heart failure, extend life, and improve health‐related quality of life in adults with heart failure with reduced ejection fraction in the United States.
- Adding empagliflozin to a standard treatment regimen for heart failure with reduced ejection fraction constitutes a cost‐effective use of health care resources, yielding health benefits for patients at an acceptable increase in costs for US Medicare and commercial health care payors.

**What Are the Clinical Implications?**

- Clinicians should consider the use of empagliflozin plus standard of care to promote avoidance of hospitalization for heart failure, reduce mortality, and improve quality of life when treating patients with heart failure with reduced ejection fraction in the United States.

Heart failure (HF) is characterized by high mortality, morbidity, and economic burden. Data from the National Health and Nutrition Examination Survey (2015–2018) suggest ≈6.0 million Americans aged 20 years or older have HF, and the prevalence of HF is expected to increase 46% from 2012 to 2030, due in large part to the aging of the population.^1^ Jackson et al report that in 2014, primary (comorbid) HF was associated with 1.1 (4.1) million emergency department visits, 1.0 (3.4) million hospitalizations, and 83 705 (230 963) deaths, with expenses for hospitalizations for primary HF alone amounting to $11 billion.^2^ Other recent studies show that in both Medicare and commercial settings, US health care expenditures associated with HF are significant and driven to a sizable extent by costs due to hospitalization.^3^, ^4^, ^5^, ^6^

Empagliflozin is a sodium‐glucose co‐transporter 2 inhibitor indicated to reduce the risk of cardiovascular death plus hospitalization for heart failure (HHF) in adults with heart failure with reduced ejection fraction (HFrEF).^7^ The addition of empagliflozin to guideline‐directed standard therapy including angiotensin‐converting enzyme inhibitors or angiotensin receptor blockers (ARB) or angiotensin receptor neprilysin inhibitors (ARNi) with mineralocorticoid receptor antagonists, β‐blockers, or diuretics was investigated in the EMPEROR‐Reduced (Empagliflozin Outcome Trial in Patients with Chronic Heart Failure and a Reduced Ejection Fraction) trial.^8^ EMPEROR‐Reduced was an event‐driven trial of empagliflozin 10 mg daily versus placebo, both added to standard therapy, in 3730 adults with New York Heart Association functional class II to IV and left ventricular ejection fraction (LVEF) of ≤40%.^8^ Empagliflozin plus standard therapy lowered the composite of cardiovascular death or HHF by 25% (hazard ratio, 0.75 [95% CI, 0.65–0.86]) and HHF by 30% (hazard ratio, 0.70 [95% CI, 0.58–0.85]) versus standard therapy.^8^

Although data suggest that empagliflozin may improve patient outcomes, understanding the economic implications of adding empagliflozin to a standard treatment regimen for HFrEF in the United States is important. The objective of this study was to develop a cost‐effectiveness model to evaluate the additional value associated with adding empagliflozin to standard of care (SoC) compared with SoC alone in patients with HFrEF in the United States.

## METHODS

### Data Availability Statement

The data and methods that support the findings of this study are available from the corresponding author upon reasonable request.

### Model Structure

**Figure 1:**
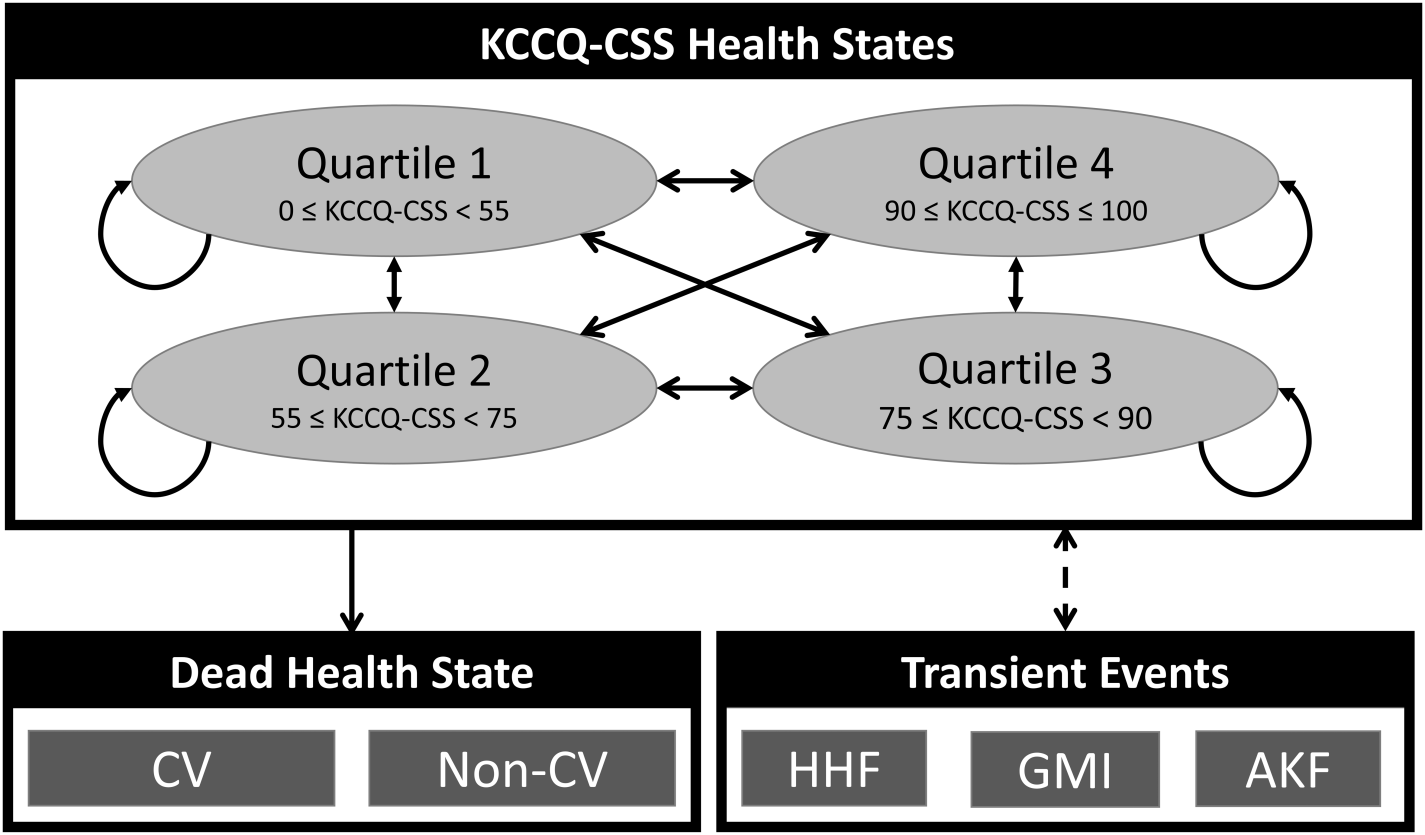
Model schematic. AKF indicates acute kidney failure; CV, cardiovascular; GMI, genital mycotic infection; HHF, hospitalization for heart failure; and KCCQ‐CSS, Kansas City Cardiomyopathy Questionnaire Clinical Summary Score.

A 5‐state Markov cohort state‐transition model (Figure 1) simulating lifetime progression of HFrEF was developed for this economic evaluation taking the perspective of a third‐party payor. Each month, a simulated patient had a chance of progressing/regressing through health states defined by Kansas City Cardiomyopathy Questionnaire Clinical Summary Score (KCCQ‐CSS) or dying. The 5 states were (1) KCCQ‐CSS quartile 1 (0≤KCCQ‐CSS<55), (2) KCCQ‐CSS quartile 2 (55≤KCCQ‐CSS<75), (3) KCCQ‐CSS quartile 3 (75≤KCCQ‐CSS<90), (4) KCCQ‐CSS quartile 4 (90≤KCCQ‐CSS≤100), and (5) death. While in any KCCQ‐CSS state, patients could be hospitalized due to worsening HF, experience a treatment‐related adverse event, or die. Distinctions were made between cardiovascular and noncardiovascular deaths.

Disease severity was captured using KCCQ‐CSS‐based health states, because it is prognostically important and a patient‐reported measure of health status compared with the traditional New York Heart Association classification.^9^, ^10^, ^11^, ^12^ Other recent economic evaluations in HFrEF and heart failure with preserved ejection fraction have used the KCCQ tool for decision‐making.^13^, ^14^, ^15^, ^16^

Using EMPEROR‐Reduced trial data, a population with characteristics equivalent to the trial population was simulated, and costs and health consequences for the trial duration and beyond were modeled. Future costs and benefits were discounted at 3% per year, consistent with the Second US Panel on Cost‐effectiveness in Health and Medicine recommendation.^17^

### Patient Population

The modeled population reflected the study population in the EMPEROR‐Reduced trial with symptomatic stable HFrEF (LVEF ≤40%), half of whom had type 2 diabetes (T2D).^8^ Patients with impaired kidney function (estimated glomerular filtration rate as low as 20 mL/min per 1.73 m^2^) were eligible for enrollment, and 20% received concomitant sacubitril/valsartan (an ARNi). A large proportion (62%) of patients were aged ≥65 years. The distribution of patients across KCCQ‐CSS quartile 1 (24.3%), quartile 2 (25.1%), quartile 3 (27.2%), and quartile 4 (23.4%) was fairly even. Detailed inclusion and exclusion criteria and patients' characteristics have been reported previously.^8^

Mean values of the EMPEROR‐Reduced intent‐to‐treat population were applied at baseline in the model. Subpopulations based on T2D history, kidney impairment, and ARNi treatment use were run through the model in scenario analyses. The model also simulated commercially insured (patients age<65 years) and Medicare (patients age≥65 years) populations separately. Within a cohort, all patients had the same mean characteristics.

We used deidentified data from a clinical trial involving human participants but did not deal with or report on any specific participants, so we did not seek institutional review board approval. However, the EMPEROR‐Reduced trial complies with the Declaration of Helsinki and the International Conference on Harmonization Good Clinical Practice guidelines and was approved by local authorities. An independent ethics committee or institutional review board approved the clinical protocol for each participating center. Participants provided written informed consent before entering the trial.

### Disease and Intervention Effects

Disease progression was modeled using monthly transition matrices between KCCQ‐CSS health states (Table S1) derived from patient‐level EMPEROR‐Reduced trial data. Independent transition matrices were used for empagliflozin plus SoC and SoC alone, and monthly state‐transition probabilities differed for 3 time periods (from baseline through month 3, month 4 through 8, and month 9 through 12). Transition matrices from the last period (month 9 through 12) were used to predict progression after the first year, assuming the probabilities remain constant long‐term. Changes in health status were more likely to occur in the first 3 months of treatment, with transitions occurring less frequently thereafter.

Monthly risk of HHF was estimated using a Poisson distribution fitted to observed data from EMPEROR‐Reduced, with treatment and current KCCQ‐CSS quartiles as covariates (Table S2). KCCQ‐CSS quartile covariates captured risk attributable to the patient's condition at a given point in time, while the treatment covariate accounted for any benefits of empagliflozin above and beyond its effect on KCCQ‐CSS. The Poisson distribution was selected to capture repeated HHF events that can happen over time. Patients treated with empagliflozin plus SoC or those in a higher KCCQ‐CSS health state (ie, lower disease burden) had a lower risk of HHF.

Event‐free survival curves were used to estimate the number of patients who die over the time horizon considered in the model. Cardiovascular death and all‐cause death were based on estimated event‐free survival curves from patient‐level EMPEROR‐Reduced data over the trial duration and extrapolated beyond over a lifetime horizon based on Weibull functions using single models, with treatment and KCCQ‐CSS quartiles as covariates (Data S1 and Table S3). Noncardiovascular death was derived from estimates of all‐cause death minus estimates of cardiovascular death. A correction was included to ensure that the monthly probability of noncardiovascular death was not smaller than that of the US general population. General population mortality was estimated from age‐ and sex‐specific US life tables (adjusted to remove cardiovascular death).^18^, ^19^

Treatment‐related adverse event risks were estimated from the rate per 100 patient‐years of adverse events for which a statistically significant difference was observed between empagliflozin plus SoC and SoC alone in the EMPEROR‐Reduced trial (Table S4). Incidence of adverse events was assumed to remain constant for all years of the model time horizon while patients remained on therapy.

Empagliflozin treatment discontinuation was based on an exponential function fitted to the Kaplan‐Meier time to treatment discontinuation curve (censored for death) from EMPEROR‐Reduced, with KCCQ‐CSS quartiles as covariates (Data S1 and Table S5), extrapolated over a lifetime horizon. Discontinuation from empagliflozin resulted in SoC treatment efficacy in the next model period and thereafter.

For subpopulation analyses, treatment effect of empagliflozin plus SoC on predicted HHF events, time to cardiovascular death, and time to all‐cause death were subpopulation‐specific; transition probabilities and time to treatment discontinuation were not differentiated by subpopulation to preserve statistical power.

Model validation was conducted by comparing predicted event rates over the median EMPEROR‐Reduced trial follow‐up period of 16 months to those observed in the trial. Predicted event rates per 100 patient‐years for total HHF, cardiovascular death, and all‐cause death were within the 95% CIs of the trial estimates for both empagliflozin plus SoC and SoC alone (Table S6).

### Costs

Direct costs were expressed in 2021 United States dollars (USD), inflated from prior years as needed using the medical component of the US consumer price index (Table 1).^20^ Costs combined commercial and Medicare rates, weighted by the age distribution (38% aged <65 years and 62% aged ≥65 years) of patients in the EMPEROR‐Reduced trial.

**Table 1:** Select Model Inputs

Input	Base‐case value	Source
Costs, $		
Empagliflozin*+SoC (monthly cost)	$737	Red Book^21^
SoC (monthly cost)	$216	Red Book^21^
HHF (acute cost per event)	$20 068	HCUP^23^
Cardiovascular death (acute cost per event)	$36 249	Obi et al^5^
GMI (acute cost per event)	$73	CMS,^24^ InHealth^25^
AKF (acute cost per event)	$10 416	HCUP^23^
Disease management (monthly cost)	$182	CMS,^24^ InHealth,^25^ AHRQ^26^
Utilities		
KCCQ quartile 1 (health state utility)	0.637	EMPEROR‐Reduced Trial analyses
KCCQ quartile 2 (health state utility)	0.720	EMPEROR‐Reduced Trial analyses
KCCQ quartile 3 (health state utility)	0.772	EMPEROR‐Reduced Trial analyses
KCCQ quartile 4 (health state utility)	0.823	EMPEROR‐Reduced Trial analyses
HHF (disutility)	−0.166	EMPEROR‐Reduced Trial analyses
GMI (disutility)	−0.024	Sullivan and Ghushchyan^30^
AKF (disutility)	−0.024	Sullivan and Ghushchyan^30^

AHRQ indicates Agency for Healthcare Research and Quality; AKF, acute kidney failure; CMS, Centers for Medicare & Medicaid Services; GMI, genital mycotic infection; HCUP, Healthcare Cost and Utilization Project; HHF, hospitalization for heart failure; KCCQ‐CSS, Kansas City Cardiomyopathy Questionnaire Clinical Symptom Score; and SoC, standard of care.

* Based on a net price of $522/month for empagliflozin 10 mg once daily.

Medication costs of empagliflozin 10 mg and SoC therapies were based on the wholesale acquisition cost (WAC) in Red Book.^21^ Indicated dosages for each product were obtained from US prescribing information. The model applied a $35 patient copayment for empagliflozin, based on middle‐tier median retail copayments for 3‐tier drug plans in the United States.^22^ Utilization of HF treatments at baseline in EMPEROR‐Reduced was used to inform cost of SoC. All modeled patients incurred SoC costs, while cost of empagliflozin accrued until treatment discontinuation. Further details are provided in Table S7.

Acute costs of managing clinical events were identified from US databases and literature reviews (Table S8). The acute cost of HHF was derived from Healthcare Cost and Utilization Project data and reflected inpatient expenditures associated with the treatment of systolic (congestive) heart failure (*International Classification of Diseases, Tenth Revision* [*ICD‐10*] code I50.2x).^23^ Terminal cost of cardiovascular death was estimated from Obi and colleagues, who estimated HF‐related inpatient costs in the 3 months preceding death as part of a retrospective study of enrollees in Medicare Advantage with Part D and commercial health plans, leveraging information contained in the Optum Research Database.^5^ Noncardiovascular death events were assumed to incur no costs and not vary by treatment allocation.

Adverse event costs considered severity. Costs associated with outpatient management of minor health issues for established patients, involving ≈10 minutes of patient contact (Healthcare Common Procedure Coding System code 92212), were assumed for genital mycotic infection.^24^, ^25^ Healthcare Cost and Utilization Project costs (*ICD‐10* code N17.x) informed inpatient expenditures for treating acute kidney failure.^23^

Monthly disease management expenses from outpatient (Healthcare Common Procedure Coding System code 92212) and emergency department (*International Classification of Diseases, Ninth Revision* [*ICD‐9*] code 428.20‐23) visits were estimated using unit costs^24^, ^25^, ^26^ and published resource use estimates (Table S9).^3^

### Utilities

To inform accrual of quality‐adjusted life years (QALY), utility weights (between 0 [death] and 1 [full health]; Table 1) were applied for each month patients spent in KCCQ‐CSS health states, based on the proportion of patients residing in each health state per cycle. One‐month disutilities for transient events (HHF, genital mycotic infection, or acute kidney failure) were applied to the proportion of modeled patients experiencing the event.

Utility weights were derived from EMPEROR‐Reduced trial patient‐level data. Trial participants' responses to the EQ‐5D‐5L questionnaire were mapped to EQ‐5D‐3L scores^27^ and converted into utility index scores by applying the appropriate value sets for the United States.^28^ A linear mixed‐effects regression model was developed, incorporating time‐varying indicators, reflecting whether a patient had an HHF within <1 month, 1 to 2 months, 2 to 4 months, and 4 to 12 months prior versus not hospitalized, as well as time‐varying levels of KCCQ‐CSS quartiles. This approach allowed estimation of utilities based on patients' current disease severity level and HHF history. The model was further adjusted for sex, age, region, and ischemic cause, and standardized baseline EQ‐5D (Table S10).

Health state‐specific utilities and a disutility associated with HHF were estimated from the linear mixed‐effects model. For KCCQ‐CSS quartile 4, the model assumed a utility weight similar to the US general population aged 60 to 69 years,^29^ which was slightly lower than the trial‐derived value for this health state; health state utility weights for quartiles 1 to 3 were adjusted by the difference between these 2 values. Published literature informed disutilities for genital mycotic infection and acute kidney failure.^30^

### Base‐Case Cost‐Effectiveness Analysis

The simulation estimated lifetime cumulative events per 100 patient‐years, life years, QALYs, and costs that accrued for empagliflozin plus SoC versus SoC alone. The incremental cost‐effectiveness ratio (ICER) was calculated as the incremental cost per QALY gained.

### Sensitivity and Scenario Analyses

Model inputs (or groups of associated inputs) were varied using plausible ranges or alternative values in deterministic sensitivity analysis to assess the robustness of model results to changes in the parameters. Treatment effect, health state utilities and disutilities, costs, and discount rates were tested. A probabilistic sensitivity analysis examined the combined effect of parameter uncertainty on the model results based on 1000 iterations using distributions reflecting parameter uncertainties: Dirichlet distributions for transition probabilities, beta distributions for utilities, and gamma distributions for adverse event rates and costs. Correlated draws from multivariate normal distributions from Cholesky decomposition of covariance matrices were used to vary coefficients of the statistical models.

A range of scenario analyses was conducted. The model was run in 6 subpopulations: patients with and without T2D, patients with (estimated glomerular filtration rate <60 mL/min per 1.73 m^2^) and without (estimated glomerular filtration rate ≥60 mL/min per 1.73 m^2^) kidney impairment, and patients with and without concomitant ARNi use at baseline. Scenarios considered differential payment rates by commercial insurers and Medicare. Short time horizons (1, 3, 5, and 10 years) were assessed.

## RESULTS

The average life expectancy was 0.19 life years more in the base‐case (5.96 versus 5.77 years), with 18% fewer HHF and 6% fewer deaths, for patients using empagliflozin plus SoC versus SoC alone (Table 2). After adjusting for quality of life, the difference in lifetime health effects was 0.19 QALYs (4.36 QALYs for empagliflozin plus SoC versus 4.17 QALYs for SoC alone). Mean lifetime costs were $95 075 per patient treated with empagliflozin plus SoC (37% in pharmacy costs and 49% in clinical event management costs) and $78 260 per patient treated with SoC alone (19% in pharmacy costs and 65% in clinical event management costs), with a difference of $16 815 per patient. Compared with SoC alone, the treatment strategy of using empagliflozin added to SoC had an ICER of $87 725/QALY.

**Table 2:** Base‐Case Analysis: Lifetime Cost‐Effectiveness Outputs

Base case	Cumulative events per 100 PY			LYs	QALYs	Cost, $	ICER, $/LY	ICER, $/QALY
	HHF	Death (CV/non‐CV)	AEs					
Empagliflozin+SoC	17.60	14.26 (9.88/4.38)	9.53	5.96	4.36	$95 075*	$88 894	$87 725
SoC	20.79	14.77 (10.34/4.43)	9.55	5.77	4.17	$78 260†		

AE indicates adverse event; CV, cardiovascular; HHF, hospitalization for heart failure; ICER, incremental cost‐effectiveness ratio; LY, life years; PY, patient‐year; QALY, quality‐adjusted life year; and SoC, standard of care.

* $35 119 in pharmacy costs, $41 660 for managing HHF or cardiovascular death, $5308 for managing AEs, and $12 988 for disease management.

^†^ $14 930 in pharmacy costs, $45 334 for managing HHF or cardiovascular death, $5420 for managing AEs, and $12 575 for disease management.

Scenario analyses resulted in ICERs for empagliflozin plus SoC versus SoC alone that, with few exceptions, fell below $150 000/QALY (Table 3). Across patient subpopulations by T2D status, kidney function, and concomitant ARNi use, ICERs ranged from $55 516 to $111 069/QALY. Using payment rates by US commercial insurers and Medicare produced ICERs of $58 616/QALY and $93 313/QALY, respectively. When evaluated over shorter periods of 1, 3, 5, and 10 years, ICERs were $227 200/QALY, $168 440/QALY, $137 363/QALY, and $103 701/QALY, respectively.

**Table 3:** Scenario Analysis: Lifetime Cost‐Effectiveness Outputs

Scenarios, Empagliflozin+SoC vs SoC	Incremental LYs	Incremental QALYs	Incremental cost, $	ICER, $/LY	ICER, $/QALY
Subpopulation					
With T2D	0.12	0.15	$13 852	$110 980	$91 703
Without T2D	0.28	0.25	$20 366	$72 784	$81 203
With kidney impairment	0.25	0.22	$17 297	$70 218	$78 591
Without kidney impairment	0.12	0.15	$16 310	$138 994	$106 133
With concomitant ARNi	0.53	0.44	$24 271	$45 619	$55 516
Without concomitant ARNi	0.12	0.14	$15 858	$129 374	$111 069
Perspective					
Commercial	0.17	0.20	$11 633	$69 528	$58 616
Medicare	0.22	0.20	$18 740	$85 388	$93 313
Time horizon					
1 y	0.00	0.02	$3959	$1 225 657	$227 220
3 y	0.03	0.05	$9218	$332 907	$168 440
5 y	0.06	0.09	$12 300	$197 875	$137 363
10 y	0.13	0.15	$15 551	$115 897	$103 701

ARNi indicates angiotensin receptor‐neprilysin inhibitor; ICER, incremental cost‐effectiveness ratio; LY, life years; QALY, quality‐adjusted life year; SoC, standard of care; and T2D, type 2 diabetes.

**Figure 2:**
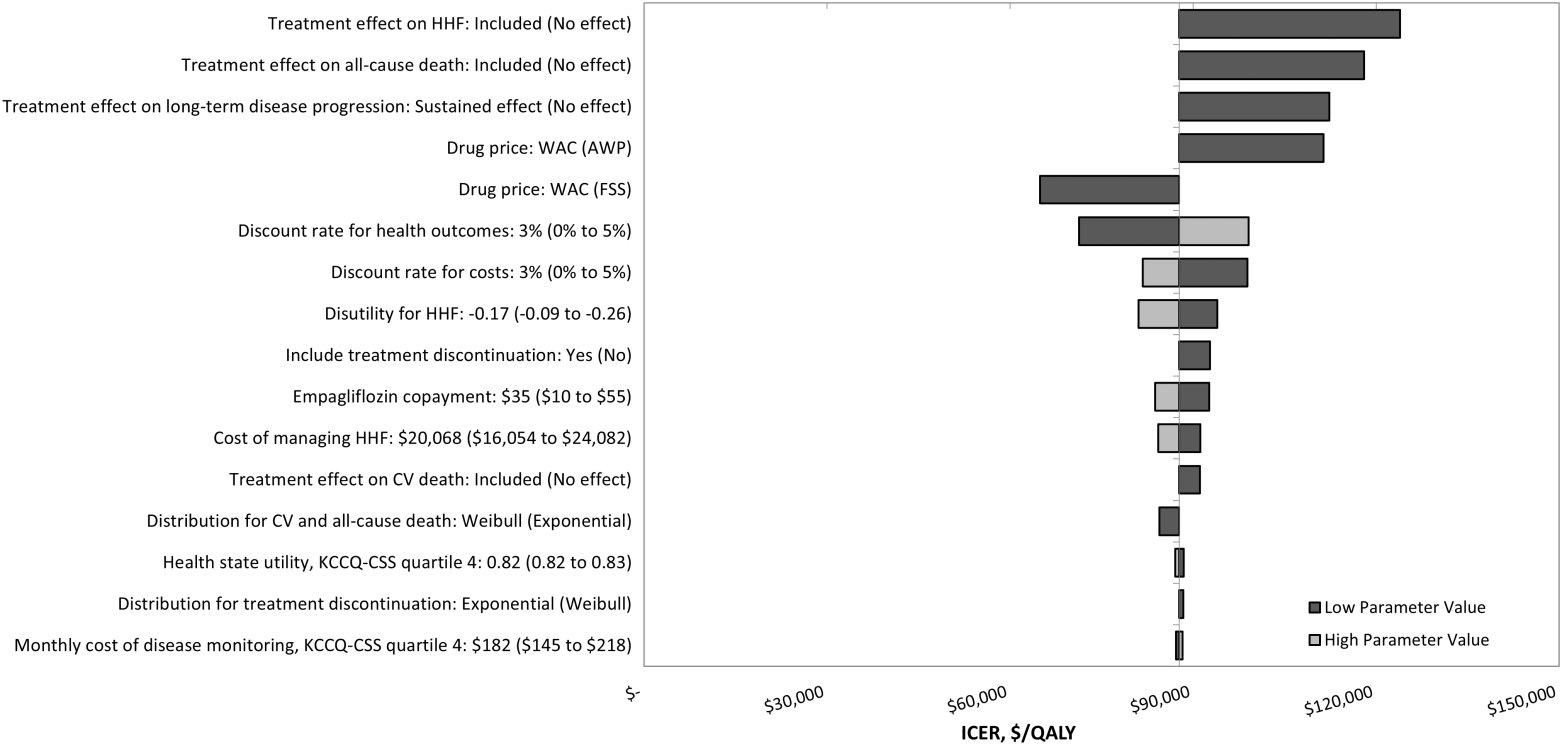
Deterministic sensitivity analysis outputs: Tornado diagram. AWP indicates average wholesale price; CV, cardiovascular; HHF, hospitalization for heart failure; ICER, incremental cost‐effectiveness ratio; KCCQ‐CSS, Kansas City Cardiomyopathy Questionnaire Clinical Symptom Score; QALY, quality‐adjusted life year; and WAC, wholesale acquisition cost.

In all deterministic sensitivity analysis scenarios presented in the tornado diagram (Figure 2), the ICERs remained <$150 000/QALY. When the treatment effect of empagliflozin plus SoC on HHF was assumed similar to SoC alone, the ICER was $123 920/QALY. In addition, assuming a treatment effect of empagliflozin plus SoC on all‐cause death resembling SoC alone yielded an ICER of $118 021/QALY. A similar assumption for treatment effect on cardiovascular death had minimal impact on the ICER ($91 118/QALY). Modeling the implications of treatment waning by assuming that the probabilities of transitioning between KCCQ‐CSS states with empagliflozin plus SoC would revert to values characteristic of SoC alone after the initial year of treatment culminated in an ICER of $112 345/QALY. If a net price of empagliflozin reflecting the average wholesale price ($633/month)^21^ or lowest reported Federal Supply Schedule price ($406/month)^31^ was used instead of WAC ($522/month), the ICER would be $111 369/QALY or $64 893/QALY, respectively.

**Figure 3:**
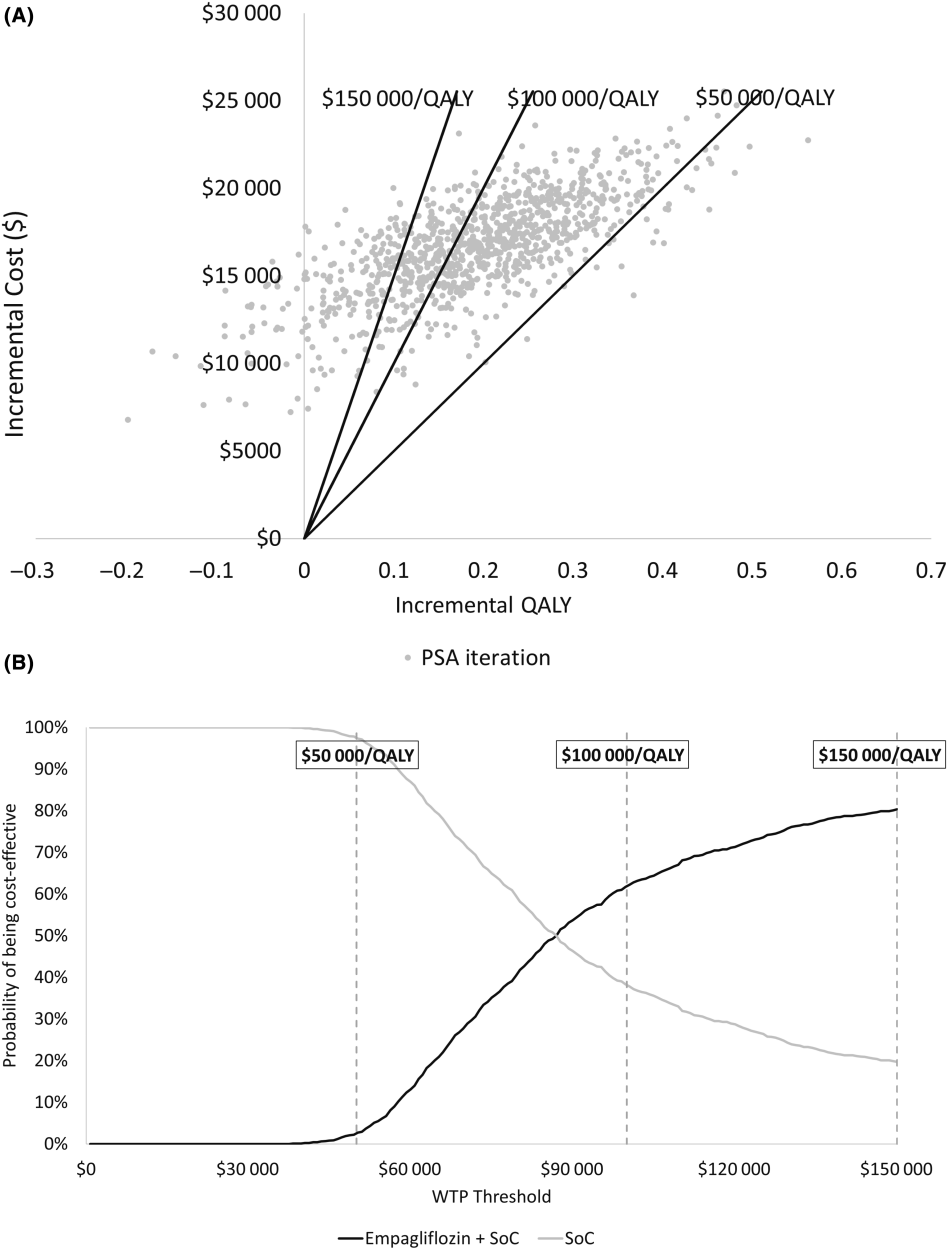
Probabilistic sensitivity analysis outputs. **A**, Cost‐effectiveness scatterplot; **B**, Cost‐effectiveness acceptability curve. PSA indicates probabilistic sensitivity analysis; QALY, quality‐adjusted life year; SoC, standard of care; and WTP, willingness‐to‐pay.

Probabilistic sensitivity analysis produced a mean ICER of $87 702/QALY, with a 3%, 62%, and 80% likelihood of empagliflozin plus SoC versus SoC alone producing ICERs below $50 000/QALY, $100 000/QALY, and $150 000/QALY, respectively. As shown in Figure 3, empagliflozin plus SoC was more costly and effective than SoC in 96.1% of iterations; in the remaining 3.9% of cases, empagliflozin plus SoC was more costly but less effective than SoC. This was attributed to uncertainty in the estimated coefficient for the empagliflozin treatment effect in the event‐free survival curves for cardiovascular and all‐cause death (Data S1 and Table S3).

## DISCUSSION

This economic evaluation found that adding empagliflozin to SoC compared with SoC alone reduced HHF and deaths and led to additional life years and QALYs at a modest cost increase in patients with HFrEF. Economic evaluations can assist decision‐makers in understanding trade‐offs associated with a new intervention that may improve clinical outcomes for patients at increased costs for health care systems, although US payors, clinicians, and patients may have different views about the value of health care. The Institute for Clinical and Economic Review, an influential independent nonprofit research institute, has published a range of recommended cost‐effectiveness thresholds ($100 000 to $150 000/QALY, although their assessments include $50 000 and $200 000/QALY) for the United States.^32^ The American College of Cardiology and American Heart Association Task Force published a cost methodology statement taking a slightly different approach and proposing categories of value.^33^ Using these benchmarks, the ICER for empagliflozin plus SoC compared with SoC alone of $87 725/QALY may be considered cost‐effective from a US payor perspective and falls well within the boundaries of intermediate value ($50 000 to $150 000/QALY) based on American College of Cardiology/American Heart Association value guidelines. However, there is a lack of national discussion about criteria to define cost‐effectiveness for policy making in the United States.

This analysis was based on the WAC of empagliflozin, an estimate of the manufacturer's list price to wholesalers/direct purchasers in the United States, which is a common benchmark in pharmacy purchasing. However, variation in price sensitivities across payors in the United States, among other factors, has manifested in a wide range of prices for brand‐name drugs, including empagliflozin. Varying the price of empagliflozin based on average wholesale price ($633/month) and the lowest reported Federal Supply Schedule price ($406/month) led to ICERs of $111 369/QALY and $64 893/QALY for empagliflozin plus SoC compared with SoC, respectively. Assuming average price concessions of 37% of the WAC ($330/month) or more would yield an ICER below $50 000/QALY, while a price of $838/month (61% higher than the WAC/base case) or less would keep the ICER below $150 000/QALY.

By contributing robust health economic evidence regarding the US cost‐effectiveness of empagliflozin plus SoC versus SoC alone for the treatment of HFrEF, this research addresses an important gap in the literature. Although recent analyses undertaken by Parikh et al and Nguyen et al sought to evaluate the cost‐effectiveness of empagliflozin for this indication in the US setting,^34^, ^35^ the model underpinning this study was comparatively more sophisticated and was informed by statistical analysis of patient‐level data gathered from EMPEROR‐Reduced. Despite marked differences in approaches, however, each study arrived at consistent conclusions about cost‐effectiveness. Additional evidence regarding the cost‐effectiveness of sodium‐glucose co‐transporter 2 inhibitors in the context of HFrEF is furnished by analyses conducted by Isaza et al^36^ and Parizo et al,^14^ which, while distinguishing themselves through both the intervention being considered (dapagliflozin) and specific details of the modeling frameworks they applied, similarly corroborated the findings of this analysis. Previous international economic evaluations with differences in model design, inputs, and assumptions, including the United Kingdom,^37^ France,^37^ Spain,^37^ and Japan,^38^ have found empagliflozin plus SoC compared with SoC alone to be cost‐effective in patients with HFrEF, considering local willingness‐to‐pay thresholds.

Recent US analyses considering the cost‐effectiveness of empagliflozin in the context of therapy for heart failure with preserved ejection fraction have also been published.^15^, ^39^ These analyses incorporate data from the EMPEROR‐Preserved trial rather than EMPEROR‐Reduced. The primary difference between the trials was the inclusion of patients with an ejection fraction of ≤40% in EMPEROR‐Reduced and >40% in EMPEROR‐Preserved. The EMPEROR‐Preserved trial population was a moderate risk population, while EMPEROR‐Reduced had enrichment for more severe HF. As a reflection of differences in patient populations, the placebo event rates also differed between the trials. There were more primary end point events per 100 patient‐years with EMPEROR‐Reduced, in line with the severity of the disease. Due to differences in the population and clinical data informing the analyses, in addition to methodological differences in the model structure, types of modeled clinical events, values of cost and utility parameters, and assumptions, analytical findings reported by Zheng et al^15^ and Cohen et al^39^ are not directly comparable to the results from this analysis.

The model has several advantages. Disease progression was modeled using a patient‐reported outcome of KCCQ‐CSS, which is an established measure of health status in HF and a secondary end point of the EMPEROR‐Reduced trial. Equations for HHF, survival, and treatment discontinuation were adjusted for patient health status in terms of KCCQ‐CSS quartile to capture the impact on health and cost outcomes. Moreover, the model incorporated US‐specific noncardiovascular death rates and costs. Finally, the model was validated by reproducing clinical event rates for empagliflozin plus SoC and SoC alone over the EMPEROR‐Reduced trial duration.

The model has some limitations that should be considered. First, KCCQ‐CSS transition matrices and clinical event risks were derived from the EMPEROR‐Reduced trial with a relatively short time horizon (median 16 months). Because the model evaluates a lifetime horizon, more evidence is needed to support the pattern of disease progression and other clinical outcomes long‐term. Second, EMPEROR‐Reduced was a multicenter, international trial, and event rates observed in US clinical practice were assumed to mirror those observed in the trial. Patients in the trial could use local guideline‐recommended HF therapies in addition to the investigative therapy, improving the likelihood of relevance to clinical practice. Third, evidence about expected time on treatment is limited; however, model results were insensitive to this parameter. Emerging real‐world evidence on treatment duration and long‐term treatment effects may allow for improved future estimates of this model parameter. Fourth, utility weights were based on the EMPEROR‐Reduced trial and applied to all modeled cohorts. The trial consisted mostly of non‐US patients. Although the responses collected in EMPEROR‐Reduced were converted to US‐specific index scores, they may have differed if they were solicited from a US population. Deterministic sensitivity analysis showed that utilities had little impact on model results; thus soliciting these values from a strictly US population would likely not alter model conclusions. Finally, results are based on US‐specific cost inputs. Although unit costs can be tailored to individual countries, generalizability to other settings outside the United States may be limited due to variations in financial and organizational structures of health care in other countries.

This pharmacoeconomic model in patients with HFrEF, based on results of the EMPEROR‐Reduced trial, suggests that adding empagliflozin to SoC compared with SoC alone leads to health benefits for patients (lower rate of HHF and improved life years and QALYs) with an acceptable increase in associated costs. Treatment with empagliflozin is likely to be cost‐effective from the perspective of US payors in managing patients with HFrEF.

## Sources of Funding

Funding for this study and article was provided by Boehringer Ingelheim Pharmaceuticals, Inc.

## Disclosures

O. S. Reifsnider, J. Ishak, P. Rakonczai, and M. Stargardter are employed by Evidera, a health care research firm that provides consulting and other research services to the biopharmaceutical and medical device industry. In these salaried positions, they work with a variety of companies and are explicitly precluded from accepting any payment or honoraria directly from those companies for services rendered. Evidera received payment from Boehringer Ingelheim Pharmaceuticals, Inc, the makers of Jardiance (empagliflozin), for collaboration on this project and article. A. Tafazzoli and S. Linden were employed by Evidera and Boehringer Ingelheim International GmbH, respectively, during the conduct of this study and creation of this article. E. Kuti is employed by Boehringer Ingelheim Pharmaceuticals, Inc.

## Supporting information

Data S1
Tables S1–S10
References 40,41
