# Supplementary material for: Cost‐Effectiveness Analysis of Empagliflozin for Treatment of Patients With Heart Failure With Reduced Ejection Fraction in the United States
Source: J Am Heart Assoc. 2024 Feb 16;13(4):e029042. doi: 10.1161/JAHA.123.029042 (PMC11010075; doi:10.1161/JAHA.123.029042)
Supplement: Supplementary file 1 — Data S1 Tables S1–S10 References 40,41 [file JAH3-13-e029042-s001.pdf]

Supplemental Material

DATA S1.

SUPPLEMENTAL METHODS

Transition Probabilities

Longitudinal measurements of Kansas City Cardiomyopathy Questionnaire Clinical Symptom Score (KCCQ-CSS) data from the EMPEROR-Reduced trial were used to estimate transition probabilities to capture disease progression in the model. KCCQ-CSS quartiles associated with the clinical severity of heart failure (HF) (symptom frequency and burden and physical limitations) were based on cut-off values of <55, <75, <90 and ≥90. KCCQ-CSS measurements collected at baseline, month 3, month 8, and month 12 were used in the analysis. Missing observations over the first 12 months (or up to the end of follow-up if the patient died or follow-up ended earlier) were handled using imputation, with missing values replaced by patients' previously observed value. Analyses consisted of estimating the proportion of patients in each health state at the current time stratified by the previous health state, with prior levels retained to allow cross-tabulation or use of this as a predictor. Long-term changes were converted to monthly transition probabilities by finding the m-root of the matrices (i.e., using the *rootm* function in R).

Modeling Recurrent HHF Events

A Poisson model with generalized estimating equations was used for recurrent hospitalization for worsening heart failure (HHF) event analysis. An auto-regressive correlation structure was assumed for repeated measures, given the trial data provided monthly follow-up for each patient. Parameterization of time in the equation was determined by plotting the observed monthly event rates for patients treated with empagliflozin plus standard of care (SoC) and SoC alone to assess

the pattern of change over time. The final equation included the time parameter and significant predictors (at $p<0.10$).

Parametric Survival Analysis

Post-hoc analyses of individual patient data from the EMPEROR-Reduced trial were performed to quantify time to cardiovascular (CV) death, all-cause death, and treatment discontinuation. The first step of the analysis involved fitting parametric distributions (exponential, Weibull, Gompertz, log-logistic, log-normal, and the generalised gamma distributions) for each event to the trial data, following the approach by Ishak and colleagues.[40] The fit for each distribution was assessed against the observed Kaplan-Meier (KM) data over the trial duration and beyond. Diagnostic plots were reviewed for a preliminary assessment of each distribution. The distribution for each event was selected considering statistical (e.g., numerical fit) and clinical considerations (e.g., clinical plausibility of the projections beyond the trial time horizon). The second step of the analysis involved developing a risk equation for each event using parametric survival analysis. The approach involved building a baseline-only equation, then introducing time-varying health states and trimming the model by removing non-significant predictors (p-value <0.10).

Table S1. Estimated Health State Transition Probabilities.

KCCQ-CSS Quartile		Mean					
		Empagliflozin + SoC			SoC		
From	To	Month 0–3	Month 4–8	Month 9+	Month 0–3	Month 4–8	Month 9+
Q1	Q1	0.796	0.910	0.918	0.835	0.904	0.929
	Q2	0.155	0.077	0.065	0.133	0.082	0.056
	Q3	0.025	0.005	0.013	0.014	0.009	0.013
	Q4	0.023	0.008	0.004	0.018	0.005	0.002
Q2	Q1	0.066	0.068	0.051	0.069	0.058	0.051
	Q2	0.708	0.840	0.881	0.720	0.850	0.867
	Q3	0.188	0.083	0.061	0.203	0.079	0.076
	Q4	0.038	0.009	0.007	0.008	0.013	0.006
Q3	Q1	0.004	0.005	0.004	0.013	0.011	0.013
	Q2	0.082	0.070	0.054	0.112	0.058	0.054
	Q3	0.772	0.848	0.868	0.743	0.859	0.871
	Q4	0.142	0.077	0.074	0.132	0.072	0.062
Q4	Q1	0.006	0.004	0.003	0.006	0.004	0.000
	Q2	0.016	0.000	0.006	0.009	0.008	0.005
	Q3	0.074	0.063	0.044	0.096	0.058	0.049

KCCQ-CSS Quartile		Mean					
		Empagliflozin + SoC			SoC		
From	To	Month 0–3	Month 4–8	Month 9+	Month 0–3	Month 4–8	Month 9+
	Q4	0.904	0.933	0.947	0.889	0.930	0.946

KCCQ-CSS, Kansas City Cardiomyopathy Questionnaire Clinical Symptom Score; Q, quartile; SoC, standard of care

Table S2. Estimated Risk Equation for HHF.

Covariate	Coefficient (SE)	p-value
Intercept	-3.347 (0.085)	0.00000
Empagliflozin treatment	-0.325 (0.097)	0.00084
KCCQ-CSS quartile 2*	-0.450 (0.103)	0.00001
KCCQ-CSS quartile 3*	-0.938 (0.114)	0.00000
KCCQ-CSS quartile 4*	-1.352 (0.142)	0.00000

Poisson model was used to estimate risk of HHF.

* Relative to KCCQ-CSS quartile 1

HHF, hospitalization for heart failure; KCCQ-CSS, Kansas City Cardiomyopathy Questionnaire Clinical Symptom Score; SE, standard error

Table S3. Estimated Risk Equations for CV Death and All-cause Death.

	CV Death		All-cause Death	
**Covariate**	**Coefficient (SE)**	**p-value**	**Coefficient (SE)**	**p-value**
Shape	1.161 (0.052)	0.000	1.204 (0.047)	0.000
Scale	0.000 (0.000)†	0.003	0.000 (0.000)‡	0.001
Empagliflozin treatment	-0.059 (0.101)	0.562	-0.044 (0.088)	0.615
KCCQ-CSS quartile 2*	-0.675 (0.129)	0.000	-0.609 (0.112)	0.000
KCCQ-CSS quartile 3*	-1.183 (0.143)	0.000	-1.161 (0.126)	0.000
KCCQ-CSS quartile 4*	-1.362 (0.148)	0.000	-1.299 (0.128)	0.000

Weibull distribution was used to inform the long-term CV death and all-cause death projections.

* Relative to KCCQ-CSS quartile 1; †Estimate has been rounded; mean (SE) = 0.00017213 (0.00005835); ‡ Estimate has been rounded; mean (SE) = 0.00016739 (0.00005102)

CV, cardiovascular; KCCQ-CSS, Kansas City Cardiomyopathy Questionnaire Clinical Symptom Score; SE, standard error

Table S4. Adverse Event Rates.

AE	Rate per 100 PYs	
	**Empagliflozin + SoC**	**SoC**
GMI[41]	1.38	0.53
AKF[41]	8.13	9.02

AE, adverse event; AKF, acute kidney failure; GMI, genital mycotic infection; PY, patient-year; SoC, standard of care

Table S5. Estimated Risk Equation for Treatment Discontinuation.

Covariate	Coefficient (SE)	p-value
Rate	0.001 (0.000)†	0.000
KCCQ-CSS quartile 2*	-0.344 (0.128)	0.007
KCCQ-CSS quartile 3*	-0.881 (0.139)	0.000
KCCQ-CSS quartile 4*	-1.083 (0.141)	0.000

Exponential distribution was used to inform treatment discontinuation.

* Relative to KCCQ-CSS quartile 1; † Estimate has been rounded; SE = 0.00008082.

KCCQ-CSS, Kansas City Cardiomyopathy Questionnaire Clinical Symptom Score; SE, standard error

Table S6. Validation of 16-Month Event Rates.

Event	Model Prediction		EMPEROR-Reduced ITT Population	
	Event Rate per 100 PY		Event Rate per 100 PY (95% CI)	
	Empagliflozin + SoC	SoC	Empagliflozin + SoC	SoC
Total HHF	15.97	21.91	15.77 (14.19, 17.31)	22.44 (20.49, 24.20)
CV death	7.10	7.80	7.55 (6.54, 8.72)	8.13 (7.08, 9.33)
All-cause death	9.41	10.20	10.06 (8.88, 11.38)	10.71 (9.49, 12.71)

CI, confidence interval; CV, cardiovascular; HHF, hospitalization for worsening heart failure; ITT, intent to treat; PY, patient-year; SoC, standard of care

Table S7. Drug Cost by Treatment Regimen (2021 USD).

**Treatment Regimen**	**Monthly Cost (utilization in EMPEROR-Reduced)**	
	**Empagliflozin + SoC**	**SoC**
SGLT2i: empagliflozin[21]	$522‡ (100%)	NA
ACEi: captopril, enalapril, lisinopril, ramipril, trandolapril* [21]	$17 (45%)	$17 (45%)
ARB: candesartan, valsartan, losartan* [21]	$19 (24%)	$19 (24%)
ARNi: sacubitril/valsartan[21]	$591 (20%)	$591 (20%)
MRA: eplerenone[21]	$43 (71%)	$43 (71%)
BB: bisoprolol, carvedilol, metoprolol, nebivolol* [21]	$45 (95%)	$45 (95%)
HCN channel blocker: ivabradine[21]	$218 (7%)	$218 (7%)
Estimated monthly cost per patient†	$737	$216

* Treatments within each class were assumed to be uniformly distributed; † Cost applied in the base case; ‡ Adjusted for a $35 copayment

ACEi, angiotensin-converting enzyme inhibitor; ARB, angiotensin receptor blocker; ARNi, angiotensin receptor neprilysin inhibitor; BB, beta blocker; HCN, hyperpolarization-activated, cyclic nucleotide-gated; MRA, mineralocorticoid receptor antagonist; NA, not applicable; SGLT2i, sodium-glucose co-transporter 2 inhibitor; SoC, standard of care; USD, United States dollar

Table S8. HHF and CV Death Cost (2021 USD).

Event	Combined*	Commercial	Medicare	ICD-10 Code
HHF[23]	$20,068	$28,487	$14,930	150.2
CV death[5]	$36,249	$68,349	$16,659	NA

* Cost applied in the base case; computed as a weighted average of Medicare and commercial costs, with weights based on the proportion of the EMPEROR-Reduced trial population that was aged 65 years or older (62%) or younger than 65 years (38%) at baseline.

CV, cardiovascular; HHF, hospitalization for heart failure; ICD, International Classification of Diseases; NA, not applicable; USD, United States dollar

Table S9. Disease Management Cost (2021 USD).

Resource	Frequency	Unit Cost			Estimated Monthly Cost per Patient		
		Combined	Commercial	Medicare	Combined*	Commercial	Medicare
Outpatient visit[3,24,25]	1.330	$110	$138	$92	$182	$220	$159
Emergency department visit[3,26]	0.008	$4,461	$4,461	$4,461			

* Cost applied in the base case; computed as a weighted average of Medicare and commercial costs, with weights based on the proportion of the EMPEROR-Reduced trial population that was aged 65 years or older (62%) or younger than 65 years (38%) at baseline.

USD, United States dollar

Table S10. Linear Mixed-effects Regression Model for Estimating Utilities.

Coefficient	Coefficient (SE)	t-value
Intercept	0.690 (0.005)	137.911
Male	0.014 (0.003)	4.251
Age ≥65 years	-0.006 (0.003)	-2.219
Region, Asia	0.000* (0.004)	0.056
Region, Latin America	0.013 (0.003)	3.796
Region, North America	0.018 (0.005)	3.853
Region, other	0.029 (0.007)	3.969
Baseline EQ-5D (standardized)	0.060 (0.002)	37.476
Ischemic HF	-0.009 (0.003)	-3.044
HF hospitalization <1 month	-0.025 (0.008)	-2.981
HF hospitalization 1≤ months <2	-0.028 (0.008)	-3.477
HF hospitalization 2≤ months <4	-0.019 (0.007)	-2.784
HF hospitalization 4≤ months <12	-0.009 (0.005)	-1.819
KCCQ-CSS quartile 2	0.082 (0.003)	24.245
KCCQ-CSS quartile 3	0.135 (0.004)	37.968
KCCQ-CSS quartile 4	0.186 (0.004)	48.813
GMI	-0.008 (0.028)	-1.416
AKF	-0.006 (0.009)	-0.906

* Estimate has been rounded; coefficient = 0.00025. AKF, acute kidney failure; GMI, genital mycotic infection; HF, heart failure; KCCQ-CSS, Kansas City Cardiomyopathy Questionnaire Clinical Symptom Score; SE, standard error
